# Base Deficit, International Normalized Ratio, and Glasgow Coma Scale (BIG) is a Predictor Tool for Survival and Mortality of Pediatric Trauma Patients

**DOI:** 10.7759/cureus.72308

**Published:** 2024-10-24

**Authors:** Liqaa Raffee, Abdel-Hameed W Al-Mistarehi, Khaled Alawneh, Khaled J Zaitoun, Shereen Hamadneh, Sohaib Bassam Mahmoud Zoghoul, Murad S Alahmad, Ayham R Alnsour, Joe Nemeth

**Affiliations:** 1 Department of Accident and Emergency Medicine, Jordan University of Science and Technology, Irbid, JOR; 2 Department of Neurosurgery, Johns Hopkins University School of Medicine, Baltimore, USA; 3 Department of Diagnostic Radiology and Nuclear Medicine, Jordan University of Science and Technology, Irbid, JOR; 4 Faculty of Medicine, Jordan University of Science and Technology, Irbid, JOR; 5 Department of Maternal and Child Health, Faculty of Nursing, Al al-Bayt University, Mafraq, JOR; 6 Department of Radiology, Hamad Medical Corporation, Doha, QAT; 7 Department of General Surgery, Hamad Medical Corporation, Doha, QAT; 8 Faculty of Medicine, Al-Balqa Applied University, Al-Salt, JOR; 9 Department of Emergency Medicine/Trauma, McGill University, Montreal, CAN

**Keywords:** big score, children, emergency, mortality, pediatrics, survival, trauma

## Abstract

Background: An accurate estimate of the survival and mortality at the initial trauma evaluation is essential to ensure appropriate triage and stratification of the patients for progressive care. One of the recognized tools for predicting mortality is the BIG Score, composed of admission base deficit, international normalized ratio (INR), and Glasgow Coma Scale (GCS). This study evaluates the BIG scale in predicting survival and mortality rates among pediatric trauma patients.

Methods: Pediatric trauma patients, aged <18 years, visiting the emergency department of a tertiary hospital in the North of Jordan from 2014 to 2019 were included. Demographic data, trauma details, and lab results were collected. The BIG score for each patient was calculated. A receiver operator characteristic (ROC) curve was generated to determine the best suitable BIG cutoff point and its probabilities.

Results: A total of 424 patients were included in this study. About two-thirds of the patients were males (n=298). The mean±SD of pH and GCS values were significantly lower among dead patients (6.0±2.5, 3.7±3.9, respectively) in comparison to alive ones (7.3±2.7, 11.5±5.5, respectively) (*p*=0.026, *p*<0.001, respectively). On the other hand, base deficit and INR values were significantly higher among dead patients (6.5±6.2, 1.9±2.5, respectively) than alive ones (1.9±3.6, 0.8±0.5, respectively), (*p*<0.001). The BIG score with a cutoff point of ≥10.0 has a high sensitivity (88.5%) and specificity (76.3%) for mortality prediction. The survival rate was correctly predicted in 100% of patients with a BIG score between 2.1 and 6. Also, the best survival predictions were seen in intubated patients (100%), followed by RTA-related trauma and ICU admission with decreasing frequency.

Conclusions: The current study has shown the added value of the BIG score as a simple and rapid tool to predict prognosis in pediatric trauma settings. The BIG score with a cutoff point of ≥10.0 is highly efficient in predicting pediatric trauma patients' mortality rates. However, the BIG score demonstrates greater accuracy in predicting survival outcomes compared to its ability to predict mortality among pediatric trauma patients.

## Introduction

Traumatic injuries are an important health concern worldwide and contribute to approximately 22.8% of the global early mortality rate [1]. Recent data indicate that trauma-related mortality continues to rise globally, with unintentional injuries, particularly road traffic accidents (RTAs), accounting for a significant portion of deaths, especially in low- and middle-income countries [2]. A common type of trauma is multiple pediatric injuries, which pose a serious threat to the lives of young children. Pediatric injuries are the primary cause of pediatric mortality and disability [3]. While global improvements in healthcare have led to reductions in early mortality from many causes, traumatic injuries, especially from RTAs, continue to be a significant cause of pediatric polytrauma, particularly in developing regions where infrastructure growth has not kept pace with necessary safety measures [4]. Another primary risk factor is interpersonal violence. The World Health Organization (WHO) reported that approximately 784,400 individuals lose their lives every day from intentional injury or interpersonal violence [5].

Jordan’s economy is developing rapidly, and traffic-related injuries now account for an increasing proportion of the country’s mortality rate (8000 deaths and 171,000 injuries in the last two decades) [6,7]. The Jordan Times reported that accidents are the second leading cause of death in the country, where the population is comprised of 43% of children or young adults [8]. Traumatic injuries are the leading cause of injury in children and young adults [6]. A later study reported that firearms-related injuries also significantly contribute to the total in Jordan [9]. A high post-trauma survival rate is linked to adequate treatment, accurate triage, and successful emergency surgery. Regardless of the trauma severity, remarkable results have been reached in pediatric traumatic cases, so it is recommended to offer the best treatment possible in the hopes of a complete recovery [10,11].

The available mortality-prediction tools are limited in their usage as the tools' variables do not help predict the mortality rate in the early phase of injury; instead, they help assess the quality after injury management [12-14]. Accurate prediction of the risk at the initial stage reduces mortality, which could probably impact the decisions related to triage, treatment, or stratification of the patients for progressive care [13,14]. One of the globally recognized tools for predicting mortality is the BIG Score which included B: Admission base deficit, I: International normalized ratio (INR), and G: Glasgow Coma Scale (GCS) [12]. The BIG score was first developed by Borgman et al. in a military setting and validated in several civilian populations [12,15-18]. The BIG score performs better than other trauma scoring systems due to its accurate prediction ratio [17]. Furthermore, GCS in the BIG Score is also an essential tool in clinical trials, as it helps determine the effective intervention in at-risk populations [19,20]. Utilizing this tool in emergency care would help in the effective and timely identification of mortality risks which consequently help manage injuries.

This study aims to expand the scope of pediatric emergency care by implementing the BIG score method in pediatric trauma patients. The rationale behind using this scale is the lack of information regarding its usage and most of the BIG score advancements, especially in the Middle East and North Africa (MENA) region. This study aims to determine the effectiveness of the BIG score in predicting mortality and survival among pediatric trauma patients. This study is the first to assess the validity of such an instrument among Jordanian pediatric traumatic patients.

## Materials and methods

Study design

To evaluate the effectiveness of the BIG score, a retrospective single-center cohort design was adopted which utilized a maintained database of pediatric traumatic patients who visited the King Abdullah University Hospital (KAUH), a tertiary hospital in the North of Jordan. Record-related trauma patients’ vital signs and patterns of suspected injuries were obtained.

Inclusion and exclusion criteria

The study included pediatric patients, aged <18 years, who visited the emergency department of KAUH from 2014 to 2019. Participants who presented with trauma, arrived at the hospital within 24 hours, and had recorded baseline values for base deficit, coagulation labs, and GCS were eligible for participation in the study. Patients intubated before being transferred to the hospital were included if a GCS was available before intubation. In comparison, those who arrived at the hospital after 24 hours of trauma were excluded. Also, patients who were dead on arrival to the ER were excluded. The study also excluded children with known co-morbidities that could influence the BIG scale components, such as coagulopathies and metabolic, cardiac, and neurologic diseases, and those with any learning or developmental disability or speech impairment. In addition, children with missing data on one or more of the BIG score components were excluded. Patients who died on the scene or before arriving at the hospital were also excluded.

Ethical consideration

All procedures performed in this study involving human participants were reviewed and ethically approved by the Institutional Review Board (IRB: 15/130/2020) of the research and ethics committee at Jordan University of Science and Technology (JUST), Irbid, Jordan. Written informed consent from the participant's legal guardian/next of kin was not required to participate in this study in accordance with the national legislation and the institutional requirements of retrospective studies. However, the patients' identities were not disclosed to preserve confidentiality.

Data collection

The database records of patients were reviewed then fed into a standardized data excel extraction form. The detailed variables included gender, age, trauma cause, type, location, and the type of transport. Along with it, their records before hospital admission helped assess the patient’s stability, hemorrhage, level of consciousness, and any pre-hospital treatment. Based on clinical variables extracted from the chart, the BIG score for each patient was calculated using the published formula (base deficit + [2.5 × INR] + [15-GCS]) [15].

Data analysis

The reviewed and collected data were analyzed using IBM SPSS Statistics for Windows, Version 25 (Released 2017; IBM Corp., Armonk, New York, United States). The patient characteristics were analyzed for surviving and dying patients using the chi-square test for categorical variables and an independent sample t-test for continuous variables. Furthermore, the area under the curve (AUC) of the receiver operating characteristic (ROC) curve was generated to determine the cutoff value for the sensitivity and specificity of the BIG score in predicting in-hospital mortality.

## Results

A total of 424 patients’ records were reviewed and included in this study. The majority of the patients were males (n = 298), while 126 were females. Patients’ demographic characteristics are shown in Table 1. Among expired cases, 74% were males, and 74.1% belonged to the childhood age group (between 2.1 and 17 years old). RTAs were the frequent cause of trauma (n = 210, 49.5%) followed by a fall (n = 187, 44.1%). Based on the injury site, 263 (62%) patients had head trauma, followed by 46 (10.8%) hands, and 43 (10.1%) patients had leg injuries. Deaths were reported with the highest frequency due to head trauma (81.5%).

**Table 1:** Patients and their injury characteristics

Variable	Alive n (%)	Expired n (%)	p-value
Gender			
Male	278 (70.0)	20 (74.1)	0.656
Female	119 (30.0)	7 (25.9)	
Age of diagnosis			
Infancy (0-2 years)	67 (16.9)	4 (14.8)	0.955
Childhood (2.1-12 years)	284 (71.5)	20 (74.1)	
Adolescence (12.1-17 years)	46 (11.6)	3 (11.1)	
Cause of trauma			
Road traffic accident (RTA)	197 (49.6)	13 (48.1)	<0.001
Blast injury	5 (1.3)	3 (11.1)	
Burn	2 (0.5)	0 (0.0)	
Falling down	182 (45.8)	5 (18.5)	
Gunshot	1 (0.3)	0 (0.0)	
Struck by a heavy object	7 (1.8)	2 (7.4)	
Unknown	3 (0.8)	4 (14.8)	
Trauma location			
Car on the road	115 (29.0)	6 (22.2)	0.185
Street	104 (26.2)	7 (25.9)	
Daycare	1 (0.3)	0 (0.0)	
Home	106 (26.7)	4 (14.8)	
Stairs	11 (2.8)	0 (0.0)	
Victim of war	5 (1.3)	1 (3.7)	
Unknown	55 (13.9)	9 (33.3)	
Isolated or polytrauma			
Isolated	249 (62.7)	4 (14.8)	<0.001
Polytrauma	143 (36.0)	20 (74.1)	
Unknown	5 (1.3)	3 (11.1)	
Location of injury at the body			
Abdominal	8 (2.7)	4 (14.8)	0.11
Leg (Ankle femur, foot, or tibia)	40 (10.0)	3 (11.1)	
Spinal cord	7 (1.7)	2 (7.4)	
Hand (distal radius, humor, or arm)	44 (11.0)	2 (7.4)	
Face	11 (2.8)	10 (37.0)	
Head	241 (60.7)	22 (81.5)	
Pelvic/hip	7 (1.7)	3 (11.1)	
Internal organ	2 (0.5)	2 (7.4)	
Unknown	14 (3.5)	0 (0.0)	

Table 2 shows a comparison of patients’ characteristics based on their survival and mortality rates. Since there was only one left against the medical advice case, it was excluded. Significant differences were observed between pH, base deficit, INR, and GCS among alive and expired patients. The means of pH values and GCS were significantly lower among expired patients in comparison to alive ones (p=0.026, p<0.001, respectively). On the other hand, base deficit and INR values were significantly higher among expired patients than alive ones, with a p-value < 0.001 for each.

**Table 2:** Comparison of age, arterial blood gases, international normalized ratio, Glasgow coma scale, and coagulation profile among alive and expired traumatic patients PT: Prothrombin Time; PTT: Partial Thromboplastin Time

Variables	Expired (27)	Alive (397)	p-Value
Age (years), mean ± SD	6.9 ± 4.4	6.4 ± 4.1	0.482
pH, mean ± SD	6.0 ± 2.5	7.3 ± 2.7	0.026
Base deficit (mmol/l), mean ± SD	6.5 ± 6.2	1.9 ± 3.6	<0.001
INR, mean ± SD	1.9 ± 2.5	0.8 ± 0.5	<0.001
GCS in ER, mean ± SD	3.7 ± 3.9	11.5 ± 5.5	<0.001
PT, mean ± SD	17.4 ± 8.4	10.6 ± 6.8	0.724
PTT, mean ± SD	34.8 ± 22.0	20.3 ± 14.6	0.117

**Figure 1:**
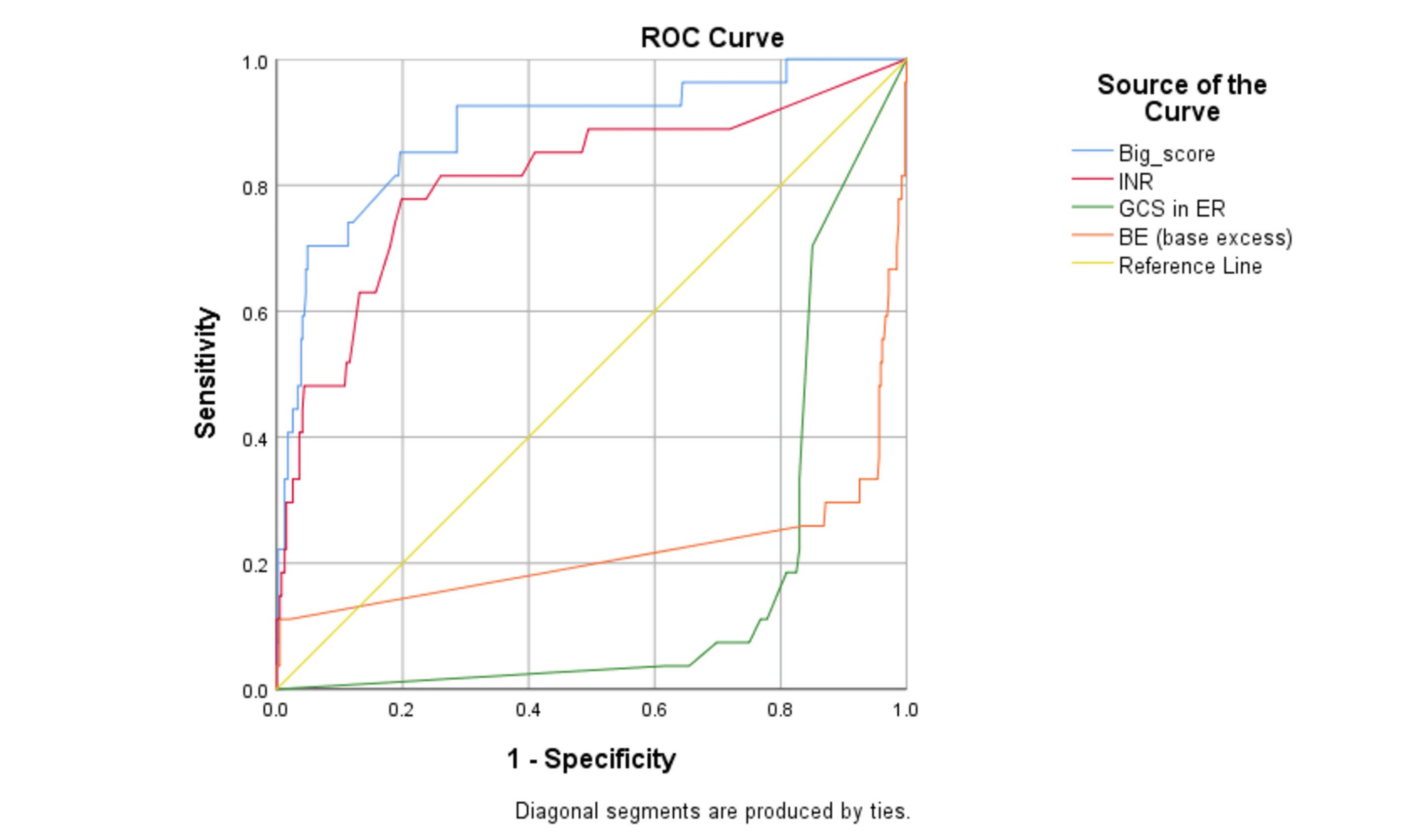
ROC curve for mortality prediction in pediatric trauma: BIG score, INR, GCS, and base excess BIG: Base Deficit, International Normalized Ratio, and Glasgow Coma Scale; GCS: Glasgow Coma Scale; INR: International Normalized Ratio

The BIG score of patients was calculated, which was used to predict the mortality rate. Figure 1 compares the ROC curves for the validity of the base deficit, INR, and GCS. The BIG score was calculated for mortality prediction. Accordingly, a BIG score of ≥10.0 has a high sensitivity (88.5%) and high specificity (76.3%) for mortality prediction. With a confidence index (CI) of 0.84-0.97, it has a negative predictive value (NPV) of 76.3% and a positive predictive value (PPV) of 88.8%. The area under the curve (AUC) value of 0.9 suggests an outstanding predicting mortality score in traumatic pediatric patients. The ROC curve further shows that the chances of mortality would increase with the increase in the BIG score. Similarly, with the increase in INR in trauma, patient mortality will increase, while, with low values of GCS and BE (base deficit excess), cases of mortality increase.

Table 3 shows a comparison of observed and predicted mortality based on BIG scores. For patients with a BIG score above 10, observed mortality was 88.5%, with the majority of deaths (n=24) occurring in this group. In contrast, for patients with a BIG score ranging from 2.1 to 6, the prediction of survival was accurate, with an observed survival rate of 100%.

**Table 3:** Comparison of observed and predicted mortality based on the BIG score* *Missing data (n = 8, with one excluded due to leaving against medical advice) BIG: Base Deficit, International Normalized Ratio, and Glasgow Coma Scale

BIG Score	Expired		Observed/ Predicted (%)	Alive		Observed/ Predicted (%)
	Predicted	Observed		Predicted	Observed	
<2	0	1	-	75	74	98
2.1-6	0	0	-	194	194	100
6.1-10	0	2	-	31	29	93.5
10.1-14	15	0	-	0	15	-
14.1-18	58	4	6.9	0	54	-
>18	42	19	45.2	0	23	-

Observed to predicted outcome of mortality is displayed in Table 4. The survival rate was correctly predicted in 99.5% of isolated injury patients (p=0.005), whereas, in multiple injury patients, the mortality outcome was correctly predicted in 27.4% of patients (p<0.001). The BIG score correctly predicted survival rates in intubated patients (100%), who needed Intensive Care Unit (ICU) treatment (96.4%), and those who had RTA-related trauma (99.3%).

**Table 4:** Patient’s predicted mortality vs actual mortality on trauma-related variables RTA: Road Traffic Accident

Variables		Observed/Predicted		Pearson chi-square, p-value (X^2^, Df)
		Mortality (%)	Survival (%)	
Type of injury	Isolated	6	99.5	0.005 (7.730, 1)
	Multiple	27.4	97.9	<0.001 (22.600, 1)
RTA-related cause of trauma		18.3	99.3	<0.001 (23.801, 1)
Admitted to ICU		32.6	96.4	0.003 (8.660, 1)
Intubated prior to arrival		37.1	100	0.029 (4.745, 1)

## Discussion

Study overview and key findings

The current study evaluated the BIG score for its mortality and survival predictability among Jordanian pediatric trauma patients. The results showed that a BIG score of ≥10.0 has a high sensitivity (88.5%) and specificity (76.3%) for mortality prediction. The factors that affected this association were as follows: INR, Blood Ph levels, base deficit, and GCS in the ER. Although most expired cases were males and belonged to childhood years of age, there were no significant statistical differences between survival and non-survival groups in age and gender (p>0.05). Pediatric patients with a history of exposure to multiple injuries, blast injury, and struck by a heavy object were significantly more likely to die. The BIG score may help predict pediatric trauma patients' survival rate more accurately than the mortality rate and better than its components alone. The BIG score showed excellent survival rate predictions regardless of injury type (isolated or multiple), injury cause, ICU admission, or pre-hospital intubation. On the other hand, these factors affected BIG score's ability to predict mortality rates.

Efficacy of the BIG score in predicting pediatric trauma outcomes

This is the first study that reveals the role of a BIG score in predicting mortality and survival rates among Jordanian children with trauma. Our results reported high sensitivity and specificity of the BIG scale are concordant with previous studies' findings [12, 17]. El-Gamasy et al. evaluated the pediatric trauma BIG score in comparison with the New Injury Severity Score (NISS) and Pediatric Trauma Score (PTS) in the emergency hospital and reported a superior sensitivity (86.7%) and specificity (71.4%) of the BIG scale over other trauma scales [17]. However, they found these values at a cutoff value of ≥12.7, while in our study, the best cutoff point was ≥10.0 for mortality prediction. There is a significant association between BIG score and mortality in pediatric patients suffering from RTA trauma who need intensive care unit treatment and intubation. The critical physiological variables encompassing the BIG score play an essential role in the trauma-related survival rate. One of the previous studies highlighted coagulopathy as the common independent predictor of mortality [21]. The traumatized patients are at greater risk of developing coagulopathy because the protein C pathway is activated, increasing fibrinolysis [22]. The components of the BIG score, including base deficit and GCS, are linked with the severity of the injury. Specifically, the base deficit is associated with shock severity and fluid requirement, whereas GCS is linked with detecting cerebral hypoperfusion and degree of brain injury [23-25].

Based on the findings of this study, it suggested that as the BIG score is performed rapidly, it would allow the physicians to communicate about the severity of the injury and recognize the degree of physiological derangement. Similar to the current study results, a study conducted by Davis et al. showed a decrease in the mortality of children with a BIG score lower than 16 [16]. The current study has shown the added value of the BIG score compared to its components alone to predict a significant prognosis.

Comparison with other trauma scoring systems

The current study findings showed that the BIG score effectively predicted survival in children with trauma, independent of injury type (isolated or multiple), injury cause, ICU admission, or pre-hospital intubation. Previous studies showed that the BIG score has better accurate predictability for blunt trauma pediatric patients than those with penetrating trauma [12, 16]. However, this data was not found in the patients’ medical records. The comparison of Brockamp et al. for the BIG Score, Trauma and Injury Severity Score (TRISS), and probability of survival (PS09) score has also corroborated the efficiency of the BIG Score for predicting mortality in the trauma patient [12]. Overall, the current study establishes the accuracy of the BIG score for evaluating the survival rate in the patients.

Based on the findings, the study suggests using the BIG score to facilitate clinicians in evaluating the children admitted to the emergency unit due to trauma. This strategy also assists in tracking the outcomes of pediatric trauma care based on the extent of the physiological derangement. Previous studies suggest the potential role of early correction of INR or base deficit and advanced aggressive care for components of the BIG score in improving the outcomes in trauma patients [15, 26]. Another study confirmed this suggestion, which showed that children without significant coagulopathy do not develop traumatic conditions [27]. Also, the BIG score could be used as a preferred tool to assess the participants’ mortality and survival chances in clinical trials. This pediatric trauma BIG score potentially can be used for research purposes in comparing the severity of illness and measuring quality assurance in trauma care. Finally, other means could be added to improve the BIG score in pediatric traumatic injury, such as instruments to evaluate perfusion, head injury, thrombo-elastography, and coagulopathy [28-30].

One of the strengths of the BIG score is waiving the need for anatomic classifications and physiologic parameters, such as vital signs. Also, it is a simple tool that could be known within minutes of emergency department arrival, as the GCS is a simple clinical assessment, whereas BE and INR could be rapidly and easily obtained by point-of-care devices and blood-gas analyzers in the emergency setting. The BIG score does not require any time-consuming variables that are not readily available in the acute phase of injury care compared with commonly used mortality-predicting TRISS.

This study highlights the simplicity, sensitivity, and specificity of the BIG score, particularly in the pediatric trauma field, making it a useful tool compared to other trauma scoring systems [17]. Our study also demonstrates the high ability of the BIG score for survival prediction among pediatric trauma patients. Also, our study demonstrates the high ability of the BIG score for survival prediction among pediatric trauma patients rather than mortality, based on objective measures (base deficit, INR, and GCS), although we did not directly compare it to immediate clinician judgment in this study. In addition, including a solid number of cases with reasonable data and details of trauma mechanisms are strengths of the study.

Limitations and recommendations for future research

However, this study acknowledges certain limitations inherent in single-center retrospective design utilizing registry data, making the external validation of the BIG score less measurable. To be fully validated, the score will need to be evaluated in a multi-center prospective study. Also, the predictive values of the BIG score were not compared with other well-known trauma scoring systems, such as TRISS, NISS, or PTS which weaken the validity of our findings. Thus, we suggest performing an accurate prospective comparison between the BIG scale and other trauma scoring systems in various settings. Another limitation is the small sample size and its restriction to a particular small region coverage. This impacts the generalizability of our findings due to the narrow socioeconomic status of the recruited sample, which limited the applicability of results to broader layers of society with different demographic and medical characteristics and social and economic conditions. Several patients were excluded due to missing one or more of the components of the BIG score, which increases the risk of selection bias. Also, some observations were missed in the patients' medical records, such as the nature of trauma as a blunt or penetrating type. However, this is part of the retrospective nature of this study. Based on our data, we could not associate the clinical decision endpoints, performed procedures and operations, and degree of resuscitation given with a threshold of the BIG score and its predictive values. However, the resuscitation and treatments were similar among the included patients, and evaluation of trauma management modalities was not one of the study objectives. In contrast, our current findings showed score-predicted survival being independent of the type of injury (isolated or multiple), trauma cause, ICU admission, and pre-hospital intubation. An additional limitation is the lack of a prospective protocol in the timeline of drawing lab data, although all labs are listed as admission labs. It is common in trauma research literature to collect laboratory data within the first 24 hours post-injury. This practice is based on the clinical imperative to promptly evaluate and monitor the immediate physiological responses of trauma patients. This timeframe aligns with standard practices in trauma care and research but does introduce potential variability in physiological assessments.

## Conclusions

This study demonstrates the high effectiveness of the BIG score as a quick and straightforward tool for survival rate prediction among pediatric trauma patients. Also, the BIG score is a good predictor for the severity of trauma and mortality rates in pediatric traumatic injuries. However, the BIG score was more accurate in survival prediction than the mortality and better than its components alone regardless of injury type, injury cause, ICU admission, and pre-hospital intubation. The BIG score has the advantage of being performed quickly based on information available shortly after admission. Thus, this score could predict clinical prognosis, determine admission criteria for trauma, and risk-stratify trauma among children. Also, the BIG score may be helpful as a research tool in future pediatric trauma studies and quality assurance research. Future prospective studies including an objective comparison of the well-known trauma score systems in various settings are recommended.
